# Practical approaches to improve vancomycin-related patient outcomes in pediatrics- an alternative strategy when AUC/MIC is not feasible

**DOI:** 10.1186/s40360-022-00606-1

**Published:** 2022-08-20

**Authors:** Kashif Hussain, Muhammad Sohail Salat, Shahzad Rauf, Manoj Rathi, Midhat Khan, Fizzah Naz, Wasif Ahmed Khan, Rahila Ikram, Gul Ambreen

**Affiliations:** 1 Department of Pharmacy, Aga Khan University Hospital, Stadium Road (Main Pharmacy), P.O Box 3500, Karachi, 74800 Pakistan; 2 Department of Pediatrics & Child Health, Aga Khan University Hospital, Karachi, Pakistan; 3 Department of Pharmacology, Faculty of Pharmacy and Pharmaceutical Sciences, University of Karachi, Karachi, Pakistan

**Keywords:** Vancomycin, Pediatrics, Vancomycin trough level, Pakistan, AUC

## Abstract

**Background:**

Anecdotal experience and studies have shown that most pediatric patients fail to reach target therapeutic vancomycin trough levels (VTLs) and required higher total daily doses (TDD). This retrospective study aims to evaluate the frequency of hospitalized children who achieved target VTLs with a vancomycin (VNCO) dosing regimen of 40-60 mg/kg/d q6h and to assess the VNCO-TDD required to attain the target and their effects on clinical outcomes in pediatric patients.

**Methods:**

After ethical approval, patients of 3 month-12 years were evaluated in this chart review study who received ≥ 3 intravenous-VNCO doses and appropriately drawn blood samples of VTLs between October 2019 to June 2020. Data were retrieved for demographic and clinical characteristics, culture reports, VNCO-regimen, subsequent steady-state VTLs, concomitant nephrotoxic medications, and serum creatinine. Clinical pharmacists made interventions in VNCO therapy and higher VNCO-TDD were used. Safety of higher vs standard daily doses and their clinical impact on duration of therapy, hospital stay, and survival were evaluated.

**Results:**

A total of 89 (39.1%) patients achieved target VTLs (SD-group). The smallest proportion (18.2%) of 2–6 years patients achieved target VTLs and reported the lowest mean value of 10.1 ± 0.2 mg/L which was a significant difference (*p* < 0.05) from all subgroups. Subtherapeutic VTLs were observed in 139 (60.9%) cases (HD-group), who received higher VNCO-TDD of 72 ± 8.9 mg/kg/d q6h to achieve the targets. Duration of therapy in culture-proven septic patients was significantly (*p* = 0.025) longer in SD-group [18.4 ± 12.2 days] than HD-group [15.1 ± 8.9 days]. Nephrotoxicity and electrolyte imbalance were comparable in groups. Length of hospital stay was significantly (*p* = 0.011) longer [median 22 (range 8–55) days] in SD-group compared to HD-group [median 16 (range 8–37) days]. Number of patients survived in HD-group were significantly (*p* = 0.008) higher than SD-group [129 (92.8%) vs 75 (84.3%)].

**Conclusion:**

Initial Vancomycin doses of 72 ± 8.9 mg/kg/day q6h are required to achieve therapeutic target in 3 months to 12 years patients. High doses are not associated with higher nephrotoxicity than reported with low doses. In addition, efficient pharmacist intervention for the use of higher VNCO-TDD may improve clinical outcomes in terms of duration of therapy, hospital stay, and survival.

## Introduction

Vancomycin (VNCO), a glycopeptide antibiotic has been used clinically for the last 70 years [1]. Its use has significantly increased over the last 40 yrs [2]. For treating invasive bacterial infections caused by ampicillin-resistant *Enterococci* and methicillin-resistant *Staphylococcus aureus* (MRSA), the drug of choice is VNCO [3]. It has several empiric indications including central venous catheter infections, septic shock, endocarditis, meningitis, skin, soft tissue and bones infections, and severe pneumonia [4].

The consensus guideline 2009 for therapeutic monitoring of vancomycin in adult patients and the Infectious Diseases Society of America (IDSA) 2011 guidelines had recommended vancomycin trough levels (VTLs) of 15–20 mg/L for treating serious infections caused by MRSA and to avoid the resistance in adult patients and by extrapolating in pediatrics [4, 5]. These guidelines were followed worldwide before the publication of new guidelines in 2020. The recent IDSA guidelines-2020 mainly focused on adults and guided pediatric doses as well and recommended designing pediatric doses to achieve an AUC of 400 mg·h/L and potentially up to 600 mg·h/L (assuming a MIC of 1 mg/L) [6].

Many pediatric studies have reported difficulty in achieving VTLs of 10 to 20 mg/L in pediatrics despite using a higher dose of 15 mg/kg IV every Q6h and most of these studies reported ≤ 50% of the cases achieved desired VTLs [4, 7–10] and a few studies suggested alternate higher starting doses [7, 10–12]. Although, the new guidelines referred to several pediatric studies and state that in pediatrics, an AUC/MIC target of 400 is more readily achievable than it is in adults and correlates to trough concentrations of 7 to 10 mg/L but recommended the higher VNCO total daily doses (TDD) of 60-80 mg/kg/d to meet the targets. In addition, VNCO half-life and clearance (CLv) are reported significantly different in pediatric patients [3, 10, 13, 14] and may lead to a wide range of levels in pediatrics and are recommended for an AUC-guided approach for dosing and monitoring. That is logistically a big challenge with the need for software and/or more than one VTL. Therefore, the possibility of using VTLs as targets is higher in many centers, especially in a resource-limited setting [15].

Therapeutic monitoring of VNCO-therapy by the clinical pharmacist is reported to have positive patient outcomes through the achievement of efficient target VTLs [16–21]. These patient-centered benefits include reduced number of adverse drug effects, shorter duration of therapy and hospital stay, and lower rates of morbidity, mortality, and antimicrobial resistance [19, 22, 23]. Achieving the therapeutic targets on time is pivotal to preventing the increasing trends of antimicrobial resistance [24].

Explanation of the subtherapeutic VTLs in pediatric, precise age difference to the corresponding VTLs with the same dosing regimens, and the age-based initial optimal daily dosing remain research gaps for investigation. No studies from Pakistan have analyzed and suggested the TDD required to achieve target VTLs in patients of different pediatric age groups. In the pediatric wards of The Aga Khan University Hospital (AKUH), Karachi, Pakistan, most of the VTLs in 3 months to 12 years children are reported as subtherapeutic following the practice of dosing 40–60 mg/kg/day in 4 divided doses and sampling for target VTLs (10–20 mg/L, assuming a MIC of 1 mg/L) is done 15–30 min prior to the scheduled 4^th^ VNCO dose.

Before practicing new guidelines with the limitations of practicing VTLs as a target instead of AUC/MIC in our setting it was a mandatory step to evaluate the existing practices and outcomes. Our study aimed to evaluate the current practices in the context of VNCO-TDD required to achieve the initial target VTLs among the admitted children in AKUH. A secondary objective was to assess the patient proportion who needed higher VNCO-TDD to reach target VTLs with the recommendation of clinical pharmacists and associated clinical outcomes with high doses in different pediatric age groups in Pakistan.

## Material and methods

### Study design and data sources

This retrospective cohort study was conducted in pediatric units of AKUH, Karachi, Pakistan from October 2019 to June 2020. Data were retrieved from hospital electronic records and patient files for clinical and demographic information, radiographic diagnostics, culture reports, VNCO dosing/frequency, appropriateness of initial sample collection time for VTLs, concomitant nephrotoxic medications, and serum creatinine (S-Cr). The sample size was calculated on achieving therapeutic trough levels, assuming that, 39–50% of the patient achieve recommended therapeutic levels, who were administered VNCO-TDD of 40–60 mg/kg/day [10, 15]. The sample size was determined to be 228 at 95% CI using PASS version 11.

### Vancomycin regimen and patients’ eligibility

All the patients from 3 months to 12 yrs were screened for inclusion, who received intravenous VNCO, and blood samples were drawn appropriately. The only first course of VNCO therapy was included for the cases exposed to VNCO therapy multiple times. We also included subjects exposed to 72 h empiric therapy and patients who were started VNCO therapy before admission to AKUH, if samples were drawn appropriately at the steady-state. All the patients who had (a) started antibiotic therapy prior to hospital admission and information about the therapy start time, sample collection time and trough values were missing (b) missing or no information about baseline renal function and/or microbiological cultures, (c) signs of acute kidney injury (AKI) prior to initiation of therapy (d) post cardiothoracic surgery, congenital heart diseases, congenital anomalies and patients on peritoneal dialysis were excluded. All the patients in the final cohort were further divided into different age groups because of expected renal function, and its ontogenesis [8]. Group-I: children of age 3–6 months, Group-II: children of > 6 month–2 years. Group-III: children of > 2 to 6 yrs and Group-IV: children of > 6 to 12 yrs [10, 11].

All the included patients initially received VNCO doses of 40-60 mg/kg/day q6h and were monitored for VTLs at 15–30 min before administering the 4th succeeding IV dose. No VNCO-regimen changes were made for the patients who achieved VTLs > 10 mg/L (assuming a MIC of 1 mg/L) with starting dose and grouped as standard-Doses (SD)-group. Clinical pharmacist intervened in increasing VNCO daily doses for patients who could not achieve target VTLs and monitored for subsequent VTL 15–30 min before 4th succeeding dose after VNCO therapy change, these patients were grouped as High-Doses (HD)-group.

### Definitions and outcome measures

Patients with a clinical presentation of septicemia, meningitis, or pneumonia, along with pathogen isolation in control cultures of blood, cerebrospinal fluid (CSF), endotracheal aspirate, or urine were defined as culture-positive sepsis. Clinical sepsis and nosocomial infection were defined as per standard definitions from the Centers for Disease Control and Prevention [25]. AKI was defined according to the AKI Network criteria (an elevated aged-based S-Cr) [26] and was assessed by changes in S-Cr level (before, during, and after the treatment). Serum potassium levels of 3.5–5.5 mEq/L and serum sodium levels of 4.5–5.5 mEq/L were considered normal [27]. The primary outcome of the study was to evaluate the mean TDD (mg/kg/day) to achieve target VTLs in pediatric patients of different age groups. Safety of higher vs standard daily doses and its clinical impact on the duration of therapy, hospital stay, and survival were evaluated as secondary outcomes.

### Statistical analysis

Statistical analysis was run using STATA version 15 (STATA Corp, Texas). The baseline characteristics of the study participants are reported by descriptive statistics. The primary outcome and secondary variables were reported in percentage and Mean (SD). Applied chi-square (χ2) test for evaluating the significance among the groups. Association among age groups and mean VTLs were analyzed with ANOVA with Bonferroni multiple comparison test. The *p*-value < 0.05 was considered statistically significant.

### Ethical approval

The study was approved by the AKUH ethical review committee and the need for informed consent was waived because of the retrospective nature of the study.

## Results

**Fig. 1:**
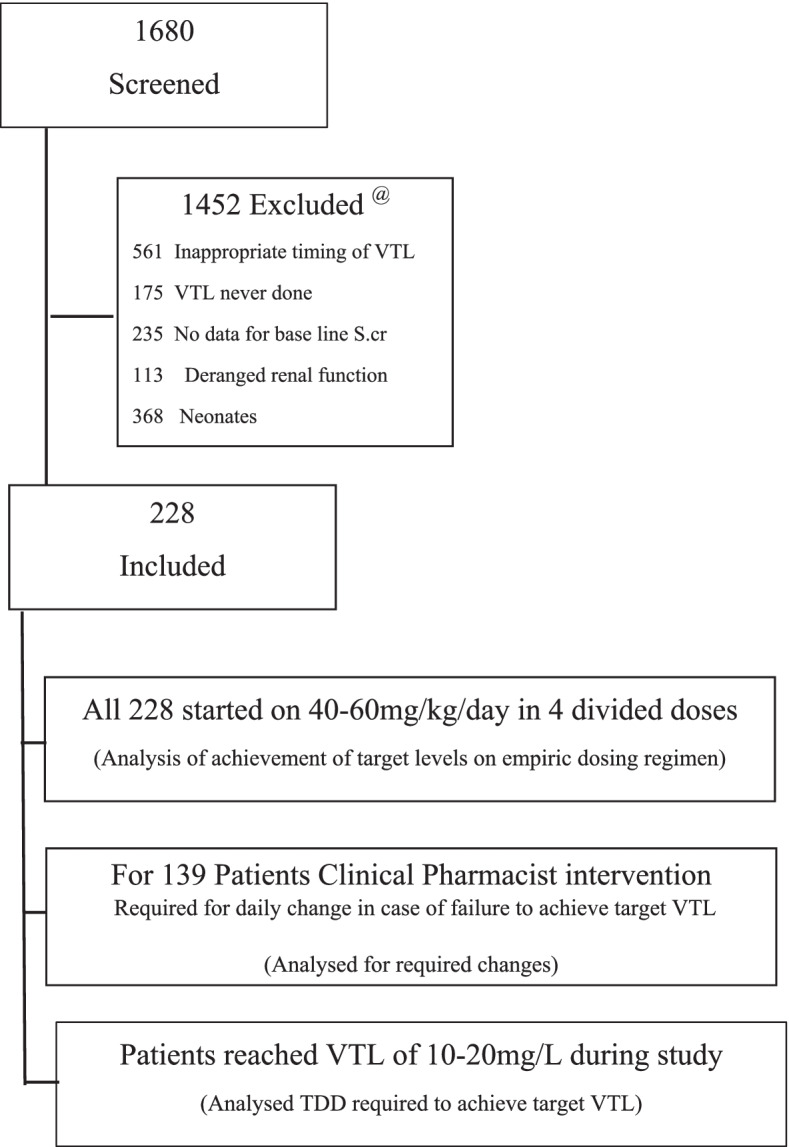
Patients Flow through the vancomycin study. ^@^ Some patients met more than one exclusion criterion. VTL = Vancomycin trough levels; S.cr = Serum creatinine; TDD = total daily dose

A total of 1680 patients were screened. Based on inclusion and exclusion criteria 1452 were excluded. Patient flow is detailed in Fig. 1. Of the 1452 patients, 228 patients aged 3 months to 12 yrs were included during the study period, who were exposed to IV VNCO-therapy, and met the study inclusion criteria. Male participants were 64.1%. Group-I:(3–6 months) had 45 (19.7%), group-II:(6 month–2 years) consisted of 70 (30.7%), Group-III:(2–6 years) included 55 (24.1%) and group-IV:(6–12 years) included 58 (25.47%) patients. The respiratory tract was the most involved system. A substantial number of patients 162 (71.1%) were admitted to the medicine department and 30 (13.2%) patients were in ICU. A total of 150 (65.8%) patients reported culture-proven infection and 78 (34.2%) received VNCO-therapy on the clinical ground (Table 1).

**Table 1:** Demographic and clinical characteristics of patients treated with vancomycin

Variables	Numbers (%)
**Total patients**	228 (100)
**Age groups**	
Group -I: children of age 3–6 months	45 (19.7)
Group -II: children of > 6 month–2 years	70 (30.7)
Group -III: children of > 2 to 6 years	55 (24.1)
Group -IV: children of > 6 to 12 years	58 (25.4)
**Gender**	
Female	82 (35.9%)
Male	146 (64.1%)
**System involved in illness/primary diagnosis /diagnosis at admission**	
CNS	21 (9.2%)
Bone and soft tissues	6 (2.6%)
Cardiac	21 (9.2%)
Colostomy	3 (1.3%)
Congenital heart disease	0 (0.0%)
Gastrointestinal	27 (11.8%)
Hematological	6 (2.6%)
Hepatic	3 (1.3%)
Metabolic disorder	12 (5.3%)
Respiratory	57 (25.0%)
Bones and soft tissues	12 (5.3%)
Soft tissue	3 (1.3%)
Sepsis	57 (25.0%)
**Unit of care/Service department**	
Pediatric ICU	30 (13.2%)
Pediatric-Medicine	162 (71.1%)
Pediatric-Surgery	36 (15.8%)
**Confirmed infection**	
No	78 (34.2%)
Yes	150 (65.8%)

Of all the study participants 89 (39.1%) could achieve the targeted VTLs of 10-20 mg/L and the rest of 139 (60.9%) cases reported subtherapeutic VTLs. Table 2 shows the detail of vancomycin doses and frequency of each group and patients’ proportions who achieved the targeted VTLs. The smallest proportion (18.2%) of children of 2–6 yrs achieved the targeted VTLs, with the lowest mean value of 10.1 ± 0.2 mg/L. The number of patients who could achieve the target VTLs in 3 to 6 months and 6 month–2 years groups were 14 (31.1%) [VTLs of 10.2 ± 0.6 mg/L] and 31 (44.3%) [VTLs of 11.3 ± 1.2 mg/L] respectively. In the 6 to 12 years group 34 (58.7%) patients achieved the comparatively higher mean VTLs of 12.4 ± 2.1 mg/L (Table 2).

**Table 2:** Vancomycin initial therapy and trough levels

Variables		3 to 6 months	> 6 month–2 years	> 2 to 6 years	> 6 to 12 years
		***N* = 45**	***N* = 70**	***N* = 55**	***N* = 58**
**dose (mg/kg/day)**	Median (range)	52.5 (45–60)	55.6 (50–60)	57.5 (55–60)	55.4 (55–60)
	Mean (± SD)	47.3 ± 13	51.1 ± 8.6	56.2 ± 6.4	54.2 ± 8.2
**interval/frequency (hourly)**	Median (range)	6	6	6	6
**Achieved target vancomycin trough levels**					
Yes- with initial dose		14 (31.1%)	31 (44.3%)	10 (18.2%)	34 (58.7%)
Mean (± SD)		10.2 ± 0.6	11.3 ± 1.2	10.1 ± 0.2	12.4 ± 2.1
No- with initial dose		31 (68.9%)	39 (55.7%)	45 (81.8%)	24 (41.3%)
Mean (± SD)		2.9 ± 1.2	3.9 ± 1.3	2.2 ± 2.0	4.2 ± 1.3

Overall 139 (60.9%) could not achieve the target VTLs and received higher vancomycin daily doses. The details of vancomycin high dose therapy and subsequent mean VTLs in each group are detailed in Table 3. One patient of two and half years reported subtherapeutic VTLs of 4.2 ± 2.0 mg/L with higher doses and two patients in the 6 to 12 years group reported supratherapeutic levels of > 20 mg/L.

**Table 3:** Post-intervention changes in Vancomycin therapy and trough levels

Variables		3 to 6 months	> 6 month–2 years	> 2 to 6 years	> 6 to 12 years
		***N* = 31**	***N* = 39**	***N* = 45**	***N* = 24**
**dose (mg/kg/day)**	Median (range)	70 (70–80)	78 (65–85)	75 (70–85)	69 (70–80)
	Mean (± SD)	71 ± 18	75 ± 9.9	73 ± 15	65 ± 18
**interval/frequency (hourly)**	Median (range)	6	6	6	6
**Achieved target vancomycin trough levels within 10-20 mg/L**					
Yes- with changed dose		31 (100.0%)	39 (100.0%)	44 (97.8%)	22 (91.7%)
Mean (± SD)		11.1 ± 0.9	12.3 ± 2.1	10.4 ± 0.2	12.8 ± 1.3
No- with changed dose		0.00 (0.0%)	0.00 (0.0%)	1 (2.2%)	02 (8.3%) ^a^
Mean (± SD)		-	-	4.2 ± 2.0	25.6 ± 3.3

^a^Supratherapeutic levels

Generally, we found a statistically significant difference among all the four age groups (*p* < 0.05) concerning the patient proportion achieving target VTLs. Multiple comparisons have shown that mean initial VTLs after standard doses were significantly different (*p* < 0.05) among all the groups except group-I (3 to 6 months) with group-III (2 to 6 years). Comparison of mean VTLs after high daily revealed that patients of all age groups had statistically significant difference (*p* < 0.05) except group-II (6 month–2 years) with group-IV (6 to 12 years) (Table 4).

**Table 4:** Comparison among different age groups

**A- Comparison based on targeted VTL achievement frequency (standard doses of 40-60 mg Q6H)**					
**Age groups**	**% Achieved targeted VTLs**	**Age groups**	**% Achieved targeted VTLs**	**RR (95% CI)**	***p*-value**
Gp-II (*n* = 70)	44.3	Gp-I (*n* = 45)	31.1	2.24 (0.91, 4.33)	** < 0.05**
Gp-I (*n* = 45)	31.1	Gp-III (*n* = 55)	18.2	4.31 (1.03, 5.11)	** < 0.001**
Gp-IV (*n* = 58)	58.7	Gp-I (*n* = 45)	31.1	3.34 (1.25, 7.34)	** < 0.05**
Gp-II (*n* = 70)	44.3	Gp-III (*n* = 55)	18.2	6.01 (3.23, 8.36)	** < 0.001**
Gp-IV (*n* = 58)	58.7	Gp-II (*n* = 70)	44.3	3.28 (1.21, 6.59)	** < 0.05**
Gp-IV (*n* = 58)	58.7	Gp-III (*n* = 55)	18.2	9.85 (5.28, 14.25)	** < 0.001**
**B- Comparison based on mean VTLs (standard doses of 40-60 mg/day Q6H)**					
**Age groups**	**VTLs mg/L** **(Mean ± SD)**	**Age groups**	**VTLs mg/L** **(Mean ± SD)**	**Means difference (95% CI)**	***p*-value**
Gp-II (*n* = 70)	11.3 ± 1.2	Gp-I (*n* = 45)	10.2 ± 0.6	1.72 (2.12, 2.97)	**0.023**
Gp-I (*n* = 45)	10.2 ± 0.6	Gp-III (*n* = 55)	10.1 ± 0.2	1.98 (1.75, 2.66)	**0.451**
Gp-IV (*n* = 58)	12.4 ± 2.1	Gp-I (*n* = 45)	10.2 ± 0.6	0.86 (- 1.28, 3.88)	**0.021**
Gp-II (*n* = 70)	11.3 ± 1.2	Gp-III (*n* = 55)	10.1 ± 0.2	2.88 (1.55, 5.55)	**0.011**
Gp-II (*n* = 70)	11.3 ± 1.2	Gp-IV (*n* = 58)	12.4 ± 2.1	2.66 (2.04, 2.80)	**0.032**
Gp-IV (*n* = 58)	12.4 ± 2.1	Gp-III (*n* = 55)	10.1 ± 0.2	1.65 (1.54, 2.65)	**0.005**
**C- Comparison based on mean VTLs (higher doses/day Q6H)**					
**Age groups**	**VTLs mg/L** **(Mean ± SD)**	**Age groups**	**VTLs mg/L** **(Mean ± SD)**	**Means difference (95% CI)**	***p*-value**
Gp-II (*n* = 39)	12.3 ± 2.1	Gp-I (*n* = 31)	11.1 ± 0.9	2.23 (2.10, 3.14)	**0.025**
Gp-I (*n* = 31)	11.1 ± 0.9	Gp-III (*n* = 45)	10.4 ± 0.2	1.88 (1.66, 3.44)	**0.041**
Gp-IV (*n* = 24)	12.8 ± 1.3	Gp-I (*n* = 31)	11.1 ± 0.9	0.82 (- 1.31, 3.44)	**0.034**
Gp-II- (*n* = 39)	12.3 ± 2.1	Gp-III (*n* = 39)	10.4 ± 0.2	2.31 (1.52, 5.75)	**0.031**
Gp-II (*n* = 39)	12.3 ± 2.1	Gp-IV (*n* = 24)	12.8 ± 1.3	2.13 (2.02, 2.17)	0.065
Gp-IV (*n* = 24)	12.8 ± 1.3	Gp-III (*n* = 39)	10.4 ± 0.2	1.48 (1.23, 2.09)	**0.026**

*VTLs* Vancomycin trough levels; Group -I: children of age 3–6 months; Group -II: children of > 6 month–2 years; Group -III: children of > 2 to 6 years; Group -IV: children of > 6 to 12 years

A comparison of patients across vancomycin treatment in SD-group and HD-groups is shown in Table 5. A significantly high number of patients were exposed to high dose vancomycin therapy in each group, who received vancomycin 72 ± 8.9 mg/kg/d Q6H, which is significantly higher than the SD-group daily dose of 55.2 ± 4.4 mg/kg/d Q6H. A significantly higher number of patients in the HD-group exposed to concomitant nephrotoxic drugs. Although more patients reported culture-proven infection in HD-group, serious Gram-positive infection was almost similar in both groups. Baseline serum creatinine was higher in SD-group, but the difference was statistically insignificant. Duration of therapy in culture-proven septic patients was significantly (*p* = 0.025) longer in SD-group [18.4 ± 12.2 days] than HD-group [15.1 ± 8.9 days]. An almost similar number of patients in both groups developed nephrotoxicity and electrolyte imbalance. Length of hospital stay was significantly (*p* = 0.011) longer [median 22 (range 8–55) days] in SD-group compared to HD-group [median 16 (range 8–37) days]. Number of patients survived in HD-group were significantly (*p* = 0.008) higher than SD-group [129 (92.8%) vs 75 (84.3%)].

**Table 5:** Comparison of demographics, comorbid conditions, and clinical characteristics across vancomycin treatment

Variables	SD-group (*n* = 89)	HD-group (*n* = 139)	*P* value
Vancomycin dose (mg/kg/d)^a^	55.2 ± 4.4	72 ± 8.9	** < 0.001**
**No. of patients in different age groups**^b^			
Group –I	14 (15.7)	31 (22.3)	** < 0.001**
Group –II	31 (34.8)	39 (28.1)	**0.027**
Group -III	10 (11.2)	45 (32.3)	** < 0.001**
Group -IV	34 (38.2)	24 (17.2)	**0.045**
**Concomitant inotropic support**^a^			
Yes	24 (21.4)	49 (35.3)	**0.045**
**Concomitant nephrotoxic drug**^a,e^			
1 Medicine	56 (62.9)	110 (79.2)	**0.008**
2 Medicines	43 (48.3)	78 (56.2)	**0.007**
3 Medicines	8 (8.9)	22 (15.8)	**0.016**
4 or more Medicines	4 (4.5)	11 (7.9)	**0.045**
**Culture proven infection**^a^			
Yes	51 (57.3)	99 (71.2)	**0.003**
**Identified Pathogen**^a^			
Coagulase-negative *Staphylococcus spp.*	16 (17.9)	22 (15.8)	0.087
MRSA	12 (13.5)	21 (15.1)	0.077
*Enterococcus spp.*	7 (7.8)	19 (13.7)	**0.045**
*Streptococci pneumoniae*	8 (8.9)	11 (7.9)	0.091
*Others*^d^	8 (8.9)	26 (18.7)	**0.004**
**Baseline serum creatinine (mg/dL)**^a^	0.55 ± 0.21	0.54 ± 0.23	0.067
**Duration of therapy (days)**^c^			
**Culture proven**			
Median (range) Mean (SD)	20 (11–35) 18.4 ± 12.2	18 (9–26) 15.1 ± 8.9	**0.025**
**Clinical sepsis**			
Median (range) Mean (SD)	6 (3–9) 5.8 ± 4.4	7 (5–7) 5.7 ± 3.2	**0.071**
**Outcome of therapy**			
Developed nephrotoxicity^b^	11 (9.9)	14 (10.1)	0.086
Developed Electrolyte imbalance^b^	16 (17.9)	22 (15.8)	0.078
Length of hospital stay^c^	22 (8–55)	16 (8–37)	**0.011**
Survival^b^	75 (84.3)	129 (92.8)	**0.008**

*SD-group* Standard dose group, *HD-group* High dose group

^a^Data presented as mean (SD); ^**b**^data presented as n (%); ^**c**^data presented as median (range)

Group -I: children of age 3–6 months; Group -II: children of > 6 month–2 years**;** Group -III: children of > 2 to 6 years; Group -IV: children of > 6 to 12 years

*MRSA* Methicillin-resistant *Staphylococcus aureus*; ^d^other isolated pathogens include Methicillin-susceptible *Staphylococcus aureus*, Beta-hemolytic *Streptococcus*, group B, and gram-negative pathogens

^e^Concomitant nephrotoxic drug includes amphotericin B, IV acyclovir, IV contrast, aminoglycoside piperacillin/tazobactam, furosemide, ACE inhibitor or ARBs and vasopressin

## Discussion

This study reports that only 39.0% of 3 months to 12 yrs old pediatric patients with normal renal function have achieved VTLs of 10–20 mg/L with the initial doses of 40–60 mg/kg q6h. Children of the 2–6 yrs age group reported the lowest frequency of reaching target VTLs and the lowest mean VTLs than all other age groups. Our results show that the highest proportion (58.7%) of children of 6 yrs to 12 years achieved the highest mean VTLs.

Clinical pharmacist intervention was needed for 61% of patients to optimize daily doses. Our results suggest that VNCO total daily doses (TDD) of 70–85 mg/kg/d in 4 divided doses can be more effective and safer to achieve the VTL > 10 mg/L in 3 months to 12 years patients. Our results are in line with previous studies [10, 13, 28–31] and almost the same daily doses are suggested by recent IDSA guidelines-2020 [6]. To the best of our knowledge, this is the first study of its kind which has evaluated the high daily vancomycin doses in pediatrics in terms of safety and clinical impacts.

The results of our study are supported by previous pediatric studies that used VNCO-TDD of 40-60 mg/kg/d and targeted VTLs of 10–20 mg/L. Frymoyer et al. [32], reported that 37% of exposed cases achieved the target levels, although Eiland et al. [7] reported a 49% success rate. Almost similar results of 39.0% are reported in another study [10]. One study reported a moderately higher number (50%) of seriously infected patients for attaining the targets [14]. Maloni et al., in their recent study, reported only 23% of pediatric patients in the ICU achieved the target [31].

Pediatric patients aged 2 to 6 yrs have the minimum likelihood to achieve the targets [10, 12, 25]. Like our study, Madigan et al., the study also reported the smallest proportion (16%-17%) of 2–6 years could attain the mean VTLs of 7.51 mg/L [13]. Another pediatric study reported almost the same trends of VTLs achievement (24.1% in 2 to 23 months, 21.7% in 2 to 6 yrs, and 33.3% in > 6 to 12 yrs) with mean VTLs of 10.3, 7.3, and 9.6 mg/L, respectively [28]. Jeffres M, et al. study found that pediatric patients aged 1 to 6 yrs mostly failed to achieve targeted VTLs [5]. Rainkie and colleagues observed that not more than 6% of children aged 1 to 6 yrs attained VTLs of 10–20 mg/L with VNCO-TDD of 60 mg/kg/d [29]. However, Benefield and colleagues reported nearly 29% and 49% of children aged 1 month to 1 yr and 6 to13 yrs respectively [33]. Moreover, the possible difference in blood sample collection time and level of sickness can explain the difference between the VTLs attainment in different studies [7, 10, 32, 34].

In our study, the lowest VTLs in 2–6 yrs patients with standard and high VNCO-TDD might be explained by the fact that age-associated discrepancies affect vancomycin clearance (CLv) as in a term neonate it is about 30 mL/minute with a half-life of nearly 7 h, that is increased to about 50 mL/minute over the age of 3 months with a shorter half-life of 4 h. The rate of CLv remains to rise with age extending to > 130 mL/minute, and half-life persists to shorten from 4 till 8 yrs age (2 to 3 h), and they reach adult’s values about 12 yrs age [3]. Hence, due to maturational variations and fluctuating renal function with the different disease conditions the patients in 2–6 yrs may require higher VNCO-TDD than other groups [10, 35–37]. Supratherapeutic VTL was reported in one case only, which can be explained by the inclusion of patients with normal renal function [13, 28, 38].

Given the data presented in this study and by previous studies [7, 10–12, 28, 32] to achieve the therapeutic targets in pediatric patients with the normal renal function it is required to initiate VNCO therapy with TDD of 70–80 mg/kg for patients of 3–6 months and 6–12 years, 65-85 mg/kg for patients of age 6 months to 2 years and 70-85 mg/kg for 2 to 6 years in 4 divided doses. Almost the same doses of 60–80 mg/kg/day are recommended by the recent IDSA guideline 2020 to achieve AUC targets of 400 to 600 mg*h/L [6]. In addition, our study suggests a specific dosing range for subgroups.

Nephrotoxicity is the major side effect related to vancomycin and the VANO-TDD in HD-group was significantly higher than SD-group. Though we followed the definition for AKI used in previous pediatric studies [26], the rate of nephrotoxicity was comparable in both groups. The need for higher VANO-TDD in HD-group might be explained by the higher number of patients with severe infections and comparatively higher inotropic and multiple antibiotics needs. For severe infections, increased renal elimination of circulating solutes is more commonly reported, which is referred to as augmented renal clearance (ARC). It can be triggered and increased by the disease itself, the inflammatory state, or therapeutic interventions [39]. Current studies suggest that ARC can lead to increased CLv, resulting in subtherapeutic VTLs, which increases the risk of antimicrobial resistance and treatment failure [6]. Therefore, in our study, timely increment of VNCO-TDD resulted in infection treatment with a shorter duration of therapy in culture-proven septic patients without causing higher nephrotoxicity than SD-group. It can be correlated with better clinical outcomes in terms of a higher number of patients discharged alive after a shorter hospital stay in HD-group [40, 41].

Limitations of this study include the shorter duration of the study. Our study was single-centered, and it did not analyze other clinical confounders that might have influenced VTLs, including weight, admission diagnosis, and malignancy. Despite a few limitations, the current study has a reasonable larger and more impactful sample size, specifically in the aspect that in our study we tried to evaluate and examine dosages in all the age-based subgroups (from 3 month to 12 yr.) that might be generally applicable. To the best of the authors' information, this is the first study to answer this study question amongst the hospitalized children in Karachi, Pakistan, and the data provides enough evidence for future clinical practices that encourage and give confidence to practitioners that usage of higher daily dosages while starting the empiric VNCO-therapy is unlikely in resulting into supratherapeutic VTLs and nephrotoxicity. Our results also suggest the significant clinical outcomes with the use of higher daily doses in terms of shorter duration of therapy, hospital stay, and survival septic patients. We also initially calculated the sample size, according to the inclusion/exclusion criteria we selected the patients with the proper sampling technique. The finding of this study in our population can provide a beginning point for randomized control trials with higher daily dosing down the line to prevent antimicrobial resistance and treatment failure [24, 42].

## Conclusion

Vancomycin given in the range of 40-60 mg/kg in daily divided doses is insufficient to achieve the therapeutic trough serum concentration for most pediatric patients aged three months to 12 yrs. The patients of 2–6 years are most likely to achieve subtherapeutic levels. This study suggests starting vancomycin doses of 72 ± 8.9 mg/kg/day in four divided doses for 3 months to 12 years patients and not resulted in higher nephrotoxicity than VNCO-TDD of 40-60 mg/kg/day. In addition, in more sick and septic patients, higher VNCO- TDD resulted in improved clinical outcomes. However, evaluation of the safety and efficacy of these proposed VNCO- TDD is needed through RCT.

## Data Availability

All data generated or analyzed during this study are included in this published article. The datasets used and/or analyzed during the current study are available from the corresponding author on reasonable request.

## Abbreviations

VNCO: Vancomycin
VTLs: Vancomycin Trough Levels
TDD: Total Daily Doses
IV: Intravenous
VNCO-TDD: Vancomycin Total Daily Doses
MRSA: Methicillin-resistant *Staphylococcus aureus*
IDSA: Infectious Diseases Society of America
AUC_24_: Area Under the Curve
MIC: Minimum Inhibitory Concentration
CLv: Vancomycin Clearance
AKI: Acute Kidney Injury
S-Cr: Serum Creatine
ARC: Augmented Renal Clearance
